# v-P_2_O_5_ micro-clustering in P-doped silica studied by a first-principles Raman investigation

**DOI:** 10.1038/s41598-019-42887-3

**Published:** 2019-05-09

**Authors:** Luigi Giacomazzi, L. Martin-Samos, A. Alessi, N. Richard, A. Boukenter, Y. Ouerdane, S. Girard, M. Valant, S. De Gironcoli

**Affiliations:** 1 Materials Research Laboratory, University of Nova Gorica, Vipavska 11c, 5270 Ajdovščina, Slovenia; 2 CNR-IOM/Democritos National Simulation Center, Istituto Officina dei Materiali, c/o SISSA, via Bonomea 265, IT-34136 Trieste, Italy; 3 SISSA, via Bonomea 265, IT-34136 Trieste, Italy; 4 Univ Lyon, UJM-Saint-Etienne, CNRS, IOGS, Laboratoire Hubert Curien UMR 5516, F-42023 St-Etienne, France; 5 CEA, DAM, DIF, F-91297 Arpajon, France

**Keywords:** Electronic properties and materials, Structure of solids and liquids

## Abstract

Synthetic vitreous silica is currently the preferred material for the production of optical fibres because of the several excellent properties of this glass, e.g. high transmission in the visible and IR domains, high mechanical strength, chemical durability, and ease of doping with various materials. For instance, fiber lasers and amplifiers exploit the light amplification properties provided by rare-earth ions employed as dopants in the core of silica-based optical fibers. The structure and composition of the nearest neighbor shell surrounding rare-earth ions in silica-based optical fibers and amplifiers have been intensively debated in the last decade. To reduce aggregation effects between rare-earth ions, co-dopants such as phosphorus and aluminium are added as structural modifiers; phosphorus-doping, in particular, has proved to be very efficient in dissolving rare-earth ions. In this work, we provide further insights concerning the embedding of P atoms into the silica network, which may be relevant for explaining the ease of formation of a phosphorus pentoxide nearest-neighbor shell around a rare-earth dopant. In particular, by means of first-principles calculations, we discuss alternative models for an irradiation (UV, *x*–, *γ*-rays) induced paramagnetic center, i.e. the so called room-temperature phosphorus-oxygen-hole center, and its precursors. We report that the most likely precursor of a room-temperature phosphorus-oxygen-hole center comprises of a micro-cluster of a few (at least two) neighboring phosphate tetrahedra, and correspondingly that the occurrence of isolated [(O-)_2_P(=O)_2_]^−^ units is unlikely even at low P-doping concentrations. In fact, this work predicts that the symmetric stretching of P=O bonds in isolated [(O-)_2_P(=O)_2_]^−^ units appears as a Raman band at a frequency of ~1110 cm^−1^, and only by including at least another corner-sharing phosphate tetrahedron, it is shown to shift to higher frequencies (up to ~40 cm^−1^) due to the shortening of P=O bonds, thereby leading to an improved agreement with the observed Raman band located at ~1145 cm^−1^.

## Introduction

The incorporation of phosphate units into the vitreous silica (*v*-SiO_2_) network is a topic of interest for applications in many research fields ranging from bioglass science to optical fibers^1,2^. For instance, Raman lasers based on P-doped silica fibers can take advantage of the larger (~3.5 times) relative Raman cross section of P_2_O_5_ (at ~1390 cm^−1^) with respect to the one of pure silica (at ~440 cm^−1^)^3,4^. Moreover, fiber lasers and amplifiers exploit the light amplification properties provided by rare-earth (RE) ions which can be included as dopants in the core of silica-based optical fibers^2^. However, since RE (e.g. Er or Yb^3+^) atoms tend to cluster, a co-dopant such as Al or P is required to dissolve the RE ions^5–10^. In particular, it has been shown that P-doping is more effective than Al in dissolving RE ions^11^. In contrast to Al-doping, P-doping could also prevent the reduction of Yb^3+^ to Yb^2+^ which is detrimental for fiber lasers applications^9,12^ as it is supposed to be responsible for the debated photodarkening processes in RE doped fibers^13–18^.

In the recent years, the composition of the nearest neighbors shell around a rare-earth ion in RE doped silica fibers (and also in other glasses^19^) has been investigated^20–22^. Still little is known concerning the formation of the nearest neighbors shell around a RE ion. One could speculate of two ways for the RE inclusion in P-doped fibers: first, the RE ion acts as an actracting center, with the P_2_O_5_ shell forming after, or secondly, through the incorporation of the RE ion into a pre-existing microscopic seed of P_2_O_5_ (a cluster or a chain made of a few phosphates) even at moderately low (≤5 mol%) P content^23^. The second hypothesis could be generalized to all P-doped silica glasses employed for optical fiber production, where P_2_O_5_ seeds could naturally be formed during the early fabrication stage.

A detailed understanding of the way P_2_O_5_ is included at microscopic levels within the silica network is highly desirable for the study and exploitation of all point defect (“centers”) related properties^24–26^ and phenomena such as photo- and radiation-darkening in optical fibers^13–17,27^. Specifically, by means of electron paramagnetic resonance (EPR) investigations it has been established that two variants of the so-called phosphorus oxygen hole center (POHC) exist in irradiated P-doped silica: the room temperature POHC (r-POHC) and the low temperature POHC (l-POHC)^25,28,29^. Griscom *et al*.^29^ proposed two models for these EPR centers: in the case of a r-POHC, a phosphate tetrahedron with two bridging and two non-bridging oxygen atoms, which share the unpaired electron. Whilst for a l-POHC: a phosphate tetrahedron with three bridging and one non-bridging oxygen atom that hosts the unpaired electron. The radiation induced generation of the these two paramagnetic centers has an almost linear dependence on the dose so that P-doped silica fibers are nowadays considered for dosimetry applications^24,30–32^.

Past investigations e.g. by means of Raman spectroscopy suggested the existence of two diamagnetic precursors^29,33^ of the above described POHC paramagnetic defects, i.e. the [(O-)_3_P(=O)]^0^ and the [(O-)_2_P(=O)_2_]^−^ tetrahedra. A Raman band at ~1330 cm^−1^ is attributed to the stretching of P=O bonds in the former phosphate tetrahedron, while a weaker Raman band at ~1150 cm^−1^ is attributed to the symmetric stretching of P=O bonds of the latter phosphate [(O-)_2_P(=O)_2_]^−^ tetrahedron^34,35^. In the literature, the frequency position of the latter Raman band in P-doped silica glasses varies between 1124 cm^−1^ to 1160 cm^−1^ for P doping concentrations up to 30 mol%^17,35–39^. While it is easily conceivable that a [(O-)_3_P(=O)]^0^ can exist, even as an isolated PO_4_ tetrahedron embedded in an otherwise ideal silica network, the formation of the [(O-)_2_P(=O)_2_]^−^ is less straightforward^29,35^ and implies an associated electron transfer/trapping mechanism i.e. through the presence of a nearby “donor defect”. Hence, the question arises as to whether such a [(O-)_2_P(=O)_2_]^−^ tetrahedron can exist as an “isolated” impurity surrounded only by SiO_4_ tetrahedra. Answering such a question from an experimental point view is very difficult, as the standard techniques to study point defects, namely EPR, OA, and luminescence, are usually not informative on structural arrangements beyond nearest neighbors, unless samples are prepared specifically for that purpose^40^. In this context, the combined use of computational techniques, in particular of the first-principles Raman and EPR spectroscopies^41,42^, represent a viable alternative to investigate the structural properties of the network surrounding a given point defect.

At variance with pure silica, the analysis of the origin of the vibrational features in P-doped silica glasses have been the object of a only limited number of investigations^34,35,38^. Initial assignments^38^ were done by interpreting the relevant features in Raman spectra of P-doped silica with the help of a vibrational mode analysis carried out for quasi-tetrahedral molecules such as POF_3_. More recent theoretical investigations could also not be so indicative, since they rely on the transferability of small cluster models^34,35,43^ or of models containing alkali elements on top of P_2_O_5_ and SiO_2_ (as in the case of bioglasses^44,45^). To the best of our knowledge an *ab-initio* calculation of suitably sized P-doped silica models is yet to be undertaken. For example, the study by Fanciulli *et al*.^35^ is based on small size clusters, which limits the amount of extractable information and leaves questions about the volume requirements necessary to host a [(O-)_2_P(=O)_2_]^−^ tetrahedron within the silica network. Furthermore, in Fanciulli *et al*.^35^ while the the calculated stretching frequency (1325 cm^−1^) of the P=O bond in the [(O-)_3_P(=O)]^0^ tetrahedron is in agreement with the experimental data, the symmetric stretching frequency of P=O bonds of the [(O-)_2_P(=O)_2_]^−^ unit is found at 1100 cm^−1^, with a ~45 cm^−1^ deviation from the experimental position of the Raman band usually attributed to it^35^. Attributing this difference simply to size effects, whilst at first seems plausible, upon further consideration, taking into account the similar size (19 and 26 atoms) of cluster models in Fanciulli *et al*.^35^, is in fact unsatisfactory.

In this work, in order to discuss the precursor models of the r-POHC, we performed first-principles calculations of the Raman spectrum for a few nm-size P-doped silica periodic models which allow for a proper description of the vibrational modes of silica glass. The present investigation, mainly based on a Raman spectra analysis, leads us to propose and validate an alternative precursor model of the r-POHC consisting of a structural unit made of (at least) two phosphate corner-sharing tetrahedra of which one is a [(O-)_2_P(=O)_2_]^−^ unit while the other a [(O-)_2_P(-O)_2_]^+^ unit^46^. In the present work we show that a corner-sharing phosphate model is able to well explain both the appearence of a band at ~1150 cm^−1^ in the Raman spectrum of P-doped silica, as well as the appearence of r-POHC centers in the irradiated glass. We also provide further evidence in favor of the attribution^35^, of the band at ~1150 cm^−1^ to the symmetric stretching of double bonds in [(O-)_2_P(=O)_2_]^−^, against to the attribution to stretching vibrations of P-O-Si or P-O-P linkages still ongoing in the literature^34,38,47–49^. Moreover our analysis provides a better understanding of how the P-doping, via [(O-)_3_P(=O)]^0^ and [(O-)_2_P(=O)_2_]^−^ units, can affect the vibrational density of states and Raman spectra of the P-doped glasses.

## Results

### P-doped models

**Figure 1:**
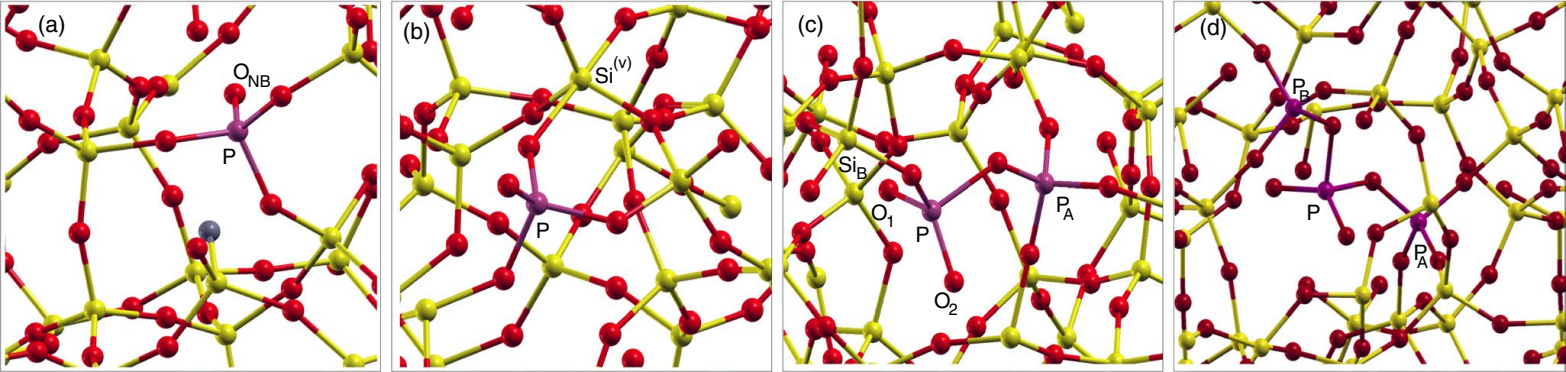
Ball and stick models of the structures investigated in this paper: (**a**) Model M1, (**b**) Model M2, (**c**) Model M3-A. By exchanging Si and P located at sites A,B one can recover the other configuration M3-B mentioned in the text. (**d**) Model M4. Silicon (yellow), oxygen (red), hydrogen (gray) and phosphorus (purple) atoms are shown.

Eight model structures have been considered in the present investigation: The original pure silica model (Model S0)^50^, a silica model containing a [(O-)_3_P=O]^0^ unit and a hydrogen passivated three-fold Si (Model M1)^46^, and the fully first-principles relaxed silica model containing a [(O-)_2_P(=O)_2_]^−^ unit (Model M2) where a penta-coordinated silicon is formed^46^. Furthermore we consider, mainly for the discussion of the localized modes of the stretching region 1000–1300 cm^−1^ (see Supplementary Fig. S1), also the not-fully relaxed model M2 which represents the ideal situation of an “isolated” [(O-)_2_P(=O)_2_]^−^ unit embedded silica (Model M2-I)^46^. Next we consider a model containing a [(O-)_2_P(=O)_2_]^−^ unit connected to a ${{\rm{PO}}}_{4}^{+}$ tetrahedron (Model M3) by sharing a corner (i.e. a bridging oxygen). As it is possible to choose such a tetrahedron in two possible ways (See. Fig. 1(c)) we distinguish them in M3-A and M3-B. Finally we consider the case (Model M4) in which the [(O-)_2_P(=O)_2_]^−^ unit is connected on both sides by ${{\rm{PO}}}_{4}^{+}$ tetrahedra (Model M4). We also performed vibrational spectra calculations for the P-doped configuration^46^ featuring a substitutional P_Si_ atom, isoelectronic to silicon, i.e. forming a [(O-)_2_P(-O)_2_]^+^ unit. The results, only marginally relevant for the present study, are included as supplementary materials (see Supplementary Fig. S2). The initial configurations used for the relaxations of models M2 and M3 have been obtained by taking advantage of a previously generated silica model featuring an ideal [(O-)_2_P(=O)_2_]^−^ unit^46^.

Concerning Model M2, its relaxation was carried by using LDA, PBE and also PBE0 functionals in order to check if the formation of the penta-coordinated silicon was an artifact of the standard (LDA/PBE) DFT functionals, as could be suggested by the rather good EPR results obtained for the isolated [(O-)_2_P(=O)_2_] model, by using hybrids functionals^35^. By contrast, we observe the formation of the penta-coordinated silicon no matter which kind of DFT approach is used as shown by the structural parameters given in Table 1.

**Table 1:** P–O_B_, O_B_-Si^v^ bond lengths and 〈P-O_B_-Si^v^〉 bond angle as obtained from the first-principles relaxation of model M2 by using LDA (PZ), GGA (PBE) and hybrid (PBE0) functionals.

	*P*–*O*_B_ (Å)	*O*_B_–*Si*^v^ (Å)	〈*P*–*O*_B_–*Si*^v^〉 (deg.)
PZ	1.507	1.896	142.30
PBE	1.547	1.900	140.49
PBE0	1.528	1.908	140.96

Si^v^ indicates the penta-coordinated silicon while O_B_ indicates the bridging oxygen between the Si^v^ and the P atom.

The starting configuration for the [(O-)_2_P(=O)_2_]^−^ unit was obtained by adding two oxygen atoms nearby a twofold P atom, thereby forming a phosphate tetrahedron^46^. The twofold P model was obtained by replacing in a silica model^51^ a twofold Si with a twofold P atom. The fact that all the relaxations carried out for the M2 model (Table 1) fail to give a stable isolated [(O-)_2_P(=O)_2_]^−^ unit, and always lead to the formation of a penta-coordinated silicon atom, is likely to indicate that the original cavity surrounding the twofold P atom is not sufficiently large to host a big structural unit such as the [(O-)_2_P(=O)_2_]^−^ unit. By contrast, as far as concerns its paramagnetic counterpart, the [(O-)_2_P(=O)_2_]^0^ (i.e. the r-POHC), the required volume is smaller and the original cavity hosting a twofold Si is large enough to allow it to relax. In fact, we have shown^46^ that such a r-POHC structural model is a real minimum by using standard DFT (LDA/PBE) techniques [see also Sec. (D)]. Concerning model M3, its relaxation was also carried out by using LDA, PBE and PBE0 functionals. It was found that the configuration shown in Fig. 1(c) is a minimum for all the applied energy functionals, with only minor variations such as those found for M2 (Table 1), thus strongly supporting the structural stability of the M3 configuration shown in Fig. 1(c). In the following, to simplify the discussion we will refer only to structural data obtained by using the LDA approximation which, in silica based materials, is known to provide reliable vibrational spectra^42^.

The P=O bonds in M1 and M2 are almost identical both showing a bond length of ~1.45 Å. The average P-O bond length in M1 and M2 are 1.55 and 1.56 Å, respectively. In the ideal configuration [(O-)_2_P(=O)_2_]^−^, M2-I, the P=O bonds have a length of 1.47 Å while the 〈O=P=O〉 is 123.1°, similarly to the structural data given for the [(O-)_2_P(=O)_2_]^−^ tetrahedron in model M3-A (Table 2). It is however worth to note that while in M2-I the bridging oxygen atoms belonging to the [(O-)_2_P(=O)_2_]^−^ tetrahedron form two symmetrically equivalent O-Si bonds with length 1.59 Å, in M3-A (and also in M3-B) the symmetry is broken as two different bonds are formed i.e. a O-Si bond (1.61 Å) and a O-P bond (1.51 Å) with its second neighboring P atom, which indeed shows an average O-P of 1.51 Å, as expected for a [(O-)_2_P(-O)_2_]^+^ tetrahedron^46^. It is also worth noting that in both M3-A and M3-B models, the P=O bonds become shorter by ~0.01 Å with respect to M2-I. The shortening of P = O bonds is even more evident in M4, where the P=O bond length is ~1.45 Å, while the angle 〈O=P=O〉 = 128.8° is slightly larger than reported for M3-A (Table 2).

**Table 2:** P=O_*I*_ bond lengths relative to the two non-bridging oxygen atoms O_1_, O_2_, and the 〈O=P=O〉 bond angle as obtained from the first-principles relaxation of model M3-A [Fig. 1(c)] by using LDA (PZ), GGA (PBE) and hybrid (PBE0) functionals.

	P=O_1_ (Å)	P=O_2_ (Å)	〈O=P=O〉 (deg.)
PZ	1.466	1.454	123.33
PBE	1.494	1.476	125.41
PBE0	1.477	1.464	125.56

### *v*-DOS and Raman results: the [(O-)_3_P=O] tetrahedron hosted in silica

**Figure 2:**
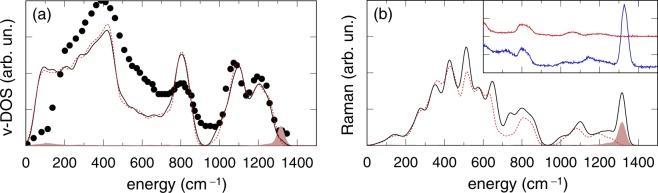
(**a**) Vibrational density of states and (**b**) HH Raman spectrum calculated for our *v*-SiO_2_ model M1 featuring a [(O-)_3_P=O] tetrahedron (solid), compared to the spectrum calculated for the original phosphorus-free *v*-SiO_2_ model (dotted/red). A gaussian broadening of 19 cm^−1^ was applied. In (**a** and **b**) the contribution of the stretching motion of the P=O bond between the P atom and its non-bridging O neighbor is also shown (shadowed). For clarity the shaded contribution in (**a**) was magnified using a factor 5. The experimental neutron density of states (discs) is displayed in (**a**) to compare peaks in the stretching region^58^. In (**b**) the inset shows the experimental Raman spectra of pure silica (solid/red) and silica P-doped with 7.4% wt (solid/blue)^73^.

In this section we investigate the vibrational density of states (*v*-DOS) and the Raman spectrum of our model M1, which features a [(O-)_3_P=O] tetrahedron embedded in pure silica, with the twofold aim of establishing the reliability of our calculations and of undertstanding which are the bands arising from the main network modifier configuration in P-doped silica^35,46^. In Fig. 2(a,b) we show the *v*-DOS and the Raman spectrum calculated for our *v*-SiO_2_ model featuring a [(O-)_3_P=O] tetrahedron, compared to the spectrum calculated for the original phosphorus-free *v*-SiO_2_ model. The *v*-DOS of the pure silica model is consistent with results from previous investigations^42,52,53^, in particular three main bands are distinguishable which are commonly attributed to oxygen rocking (0 to ~550 cm^−1^), bending (~550 to ~920 cm^−1^) and stretching (above ~920 cm^−1^) motions^53,54^. The Raman spectrum of pure silica is known to contain two sharp features called D_1_ and D_2_ at 490 and 605 cm^−1^ respectively, and attributed to the breathing modes of four- and three-membered rings. In our pure silica model, such ring modes occur at ~510 and ~610–630 cm^−1^ and are mainly responsible for the peaks located at ~515 and 628 cm^−1^ in Fig. 2(b). The *v*-DOS of the pure silica model in the stretching region presents a high-frequency doublet^55^ with peaks at ~1090 cm^−1^ and ~1200 cm^−1^ which are then reflected in analogous Raman features at 1082 cm^−1^ and at ~1193 cm^−1^. The first lower frequency stretching band shows a ~20 cm^−1^ overestimation with respect to the corresponding experimental feature, while the higher strenching band only slightly underestimates (~7 cm^−1^) the experimental band. In addition the frequency position of the minimum between the two stretching bands is overestimated in our calculation by ~15 cm^−1^ with respect to the experimental data (1142 cm^−1^).

The *v*-DOS of the model M1 is almost identical to that of the pure *v*-SiO_2_ model [Fig. 2(a)]. The presence of the [(O-)_3_P=O] tetrahedron leads to minor effects that are noticeable at the band edges of the stretching region (950 to 1350 cm^−1^). The peak located at approximately 800 cm^−1^, mainly related to the silicon motion^54^, exhibits a slight, but non-negligeable, decrease in height and a slight increase in width in the P-doped model owing to the presence of rather localized P-induced modes at ~740 cm^−1^. An increase of the *v*-DOS at the left side of the silicon peak is expected because the P atomic mass is slightly larger than Si atomic mass. Between ~400 and 700 cm^−1^ other minor differences arise between the *v*-DOS of the P-doped model and the pure silica. These are likely to be related to the P-doping and are reflected in the increased Raman intensity at ~512 cm^−1^ and ~647 cm^−1^. A direct inspection (Supplementary Fig. S3) of the vibrational modes at close frequencies reveals that the Raman intensity at ~650 cm^−1^ partly arises from the antisymmetric bending vibrational mode of the [(O-)_3_P=O] tetrahedron^56,57^, while a kind of symmetric bending of the [(O-)_3_P=O] tetrahedron appears to substantially contribute to the intensity around ~510 cm^−1^. It is worth noting that several modes in the range ~630 to 750 cm^−1^ do show the antisymmetric bending vibrational modes of the [(O-)_3_P=O] tetrahedron, thereby offering at least one explanation for the emergence of the feature at ~720 cm^−1^ in the experimental Raman spectrum of P-doped silica^39^.

In the oxygen rocking region (frequencies smaller than ~400 cm^−1^) both the *v*-DOS and Raman spectrum of the P-doped model only differ slightly from the corresponding spectra of the pure silica model (Fig. 2). In summary, the most evident difference between the Raman spectra of the pure silica model and the model M1 is the peak located at ~1320 cm^−1^, which originates from the stretching mode of the P=O bond, in good agreement with the experimental data^35^. The results shown in Fig. 2 are consistent with previous theoretical and experimental investigations^34–36,58^ and demonstrates the capability of the adopted modelling approach to properly describe the vibrational spectra of P-doped silica.

### *v*-DOS and Raman spectra of an isolated [(O-)_2_P(=O)_2_]^−^ tetrahedron hosted in silica

**Figure 3:**
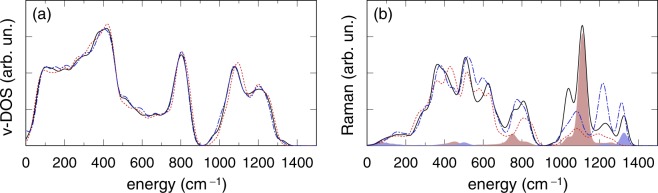
(**a**) *v*-DOS and (**b**) HH Raman spectrum of the model M2 (dot-dashed/blue), of the “ideal” silica configuration, model M2-I (solid) and of the original pure silica model (dotted/red). In (**b**) Raman intensity obtained after projection on the P=O symmetric stretching (shaded pink) and asymmetric stretching (shaded blue) modes is also shown for model M2-I.

The *v*-DOS and Raman spectrum of the P-doped model M2, featuring a phosphate tetrahedron linked to a penta-coordinated silicon [Fig. 1(b)], are shown in Fig. 3. In the Raman spectrum of the P-doped model M2, the P=O stretching mode occurs at 1314 cm^−1^ close to the corresponding feature of Fig. 2. However the Raman intensity calculated for the model M2 in Fig. 3(b) has a minimum at ~1150 cm^−1^, where the P=O stretching modes of the [(O-)_2_P(=O)_2_]^−^ unit should appear according to previous assignments^35^. Furthermore, a rather intense peak appears at about 1220 cm^−1^ which has never been experimentally observed and, from a direct inspection of the vibrational modes, can be attributed to the displacements of oxygen atoms of the penta-coordinated silicon connected to the phosphate unit (Supplementary Fig. S4).

In Fig. 3(a,b) we also show *v*-DOS and the Raman intensity as calculated for our model M2-I which represents the ideal case of an isolated [(O-)_2_P(=O)_2_]^−^ tetrahedron. It should be noted that the global shape of the Raman spectrum up to ~900 cm^−1^ is very similar to the one calculated for the fully relaxed M2 model shown in Fig. 3(b), thus supporting the reliability of the vibrational modes calculated for the M2-I configuration. Furthermore, it is worthwile noting that between ~1030 and ~1140 cm^−1^ the *v*-DOS of M2-I and M2 are almost identical Fig. 3(a). The stretching band of the Raman spectrum calculated for model M2-I is however rather different with respect to the one calculated for model M2, in particular an intense and rather sharp Raman band is visible at 1111 cm^−1^ which, on the basis of our analysis, can be attributed to the symmetric stretching of P=O bonds of the [(O-)_2_P(=O)_2_]^−^ tetrahedron. The asymmetric stretching of P=O bonds gives rise to a smaller (less intense by an order of magnitude) feature appearing at 1325 cm^−1^. In order to establish the reliability of the frequency position of the symmetric stretching as visible in Fig. 3(b), we performed a Raman calculation and mode analysis using the same cluster model used by Fanciulli *et al*.^35^, i.e. the O_2_P(OSiO_3_H_3_)_2_. For such a cluster model we found the symmetric stretching of P=O to occur mainly at 1117 cm^−1^ (there is a minor component, about four times less intense, also at 1150 cm^−1^, see Supplementary Fig. S5). The latter frequency slightly (17 cm^−1^) overestimates the B3LYP calculation of Fanciulli *et al*.^35^ which provides a value of 1100 cm^−1^. Hence the frequency (1111 cm^−1^) of the symmetric stretching of P=O bonds as calculated for the M2-I configuration can be regarded as in reasonable agreement with calculations based on the cluster model^35^.

### Raman spectra of P-doped silica models including a [(O-)_2_P(=O)_2_]^−^ unit

**Figure 4:**
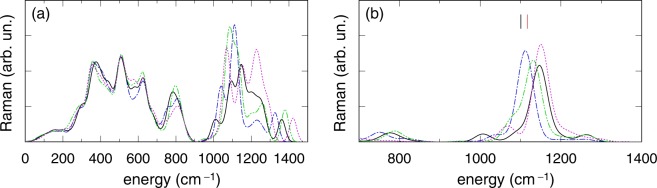
(**a**) HH Raman spectrum calculated for the models M4 (dotted/purple), M3-A (solid), M3-B (double-dot-dashed/green) and M2-I model (dot-dashed/blue). In (**b**) the HH Raman spectrum of models M2-I,M3,M4 is analyzed by showing the contribution of the symmetric stretching of the two non-bridging O atoms [same symbols/colors as in (**a**)]. Segments located at 1101 and 1117 cm^−1^ indicate the position of the symmetric stretching in the calculations of Fanciulli *et al*.^35^ (black) as well as of the present study (red).

In Fig. 4(a) we compare the Raman spectrum as calculated for the P-doped silica models M3-A, M3-B and M4, which include corner-sharing phosphate units, and M2-I which features an isolated [(O-)_2_P(=O)_2_]^−^ phosphate unit. The spectra are very similar up to 900 cm^−1^ with minor differences at about 570 cm^−1^ and 780 cm^−1^ which could be a consequence of the higher P content in M4 and M3 with respect to M2-I. Above 900 cm^−1^, the Raman spectrum calculated for the M3-A configuration shows a dominant band in the stretching region with a peak at 1148 cm^−1^ which can be attributed to the symmetric stretching of P=O bonds in the [(O-)_2_P(=O)_2_]^−^ unit [Fig. 4(b)]. It is worth noting, however that in comparison to the M2-I model, the presence of a second phosphate tetrahedron, besides the [(O-)_2_P(=O)_2_]^−^ unit, in models M3-A and M3-B leads to a broadening of the Raman intensity around the symmetric stretching frequencies of P=O bonds. The broadening is even more noticeable in M4, for which the Raman spectrum shows remarkably broad intensity from ~1050 up to 1250 cm^−1^. In particular, at variance with model M2-I, no sharp feature (i.e. FWHM ≤ 50 cm^−1^) is observed for models M3 and M4, consistently with the rather large width (FWHM ~ 100 cm^−1^) of the band at ~1150 cm^−1^ ^35^. A Raman peak which is due to the asymmetric stretching of the P=O bonds in the [(O-)_2_P(=O)_2_]^−^ unit appears at ~1300–1400 cm^−1^, but for all the examined models (M2-I, M3-A, M3-B, M4) its intensity is considerably smaller when compared to the bands in the range 1100–1200 cm^−1^.

In Fig. 4(b) we show the Raman intensity obtained after the projection of vibrational modes onto the symmetric stretching of P=O bonds. The symmetric stretching of P=O bonds is a strongly Raman active mode as can be inferred by the cluster calculations^35^ or by Fig. 3. Figure 4(b) displays a tendency to shift to higher frequencies with increasing number of corner-sharing phosphate tetrahedra connected to the [(O-)_2_P(=O)_2_]^−^ unit, with a maximal shift of 38 cm^−1^ registered between peaks of projections done for M4 (two PO_4_) and for M2-I (zero PO_4_) models.

### EPR results: comparing the isolated vs corner-sharing phosphate models of the r-POHC in silica

The M2, M3, and M4 models discussed in the previous section are supposed to be precursors of the paramagnetic center known as r-POHC, which is observed in irradiated P-doped silica samples^25,29^. It should be noted that the estimated concentration of POHC centers is much lower, a few orders of magnitude, than the likely concentration of its precursors^25^. In this section we discuss the EPR parameters calculated for the paramagnetic configurations obtained by properly charging the M2, M3, and M4 configurations. This is done with the main purpose of checking that all these structural models are indeed reliable precursors of the r-POHC, and not with the purpose of establishing what is the most realistic model for the r-POHC. In our recent work^46^, it was suggested that by removing one electron from M2, and subsequently relaxing the structure, it is possible to generate a model of the isolated r-POHC. Similarly, we performed a structural optimization of model M3 which was previously positively charged (q = +1). The first-principles relaxation of M3 in the q = +1 charged state does not lead to any change in the bonding (no breaking or forming new bonds) and the relaxed configuration shows structural similarity with the r-POHC as obtained from M2^35^. In the r-POHC models, as represented by the charged M2 and M3, M4 models, the P=O bond length is slightly longer (increase by ~0.02–0.03 Å) than found for the P=O in the corresponding precursor model (~1.49 Å). The O=P=O angle becomes narrower (it decreases by ~20° down to ~105°) in agreement with Fanciulli *et al*.^35^ The change in the O=P=O angle is then reflected by a decrease of ~3% in the volume of the [(O-)_2_P(=O)_2_] tetrahedron when it becomes a r-POHC.

**Figure 5:**
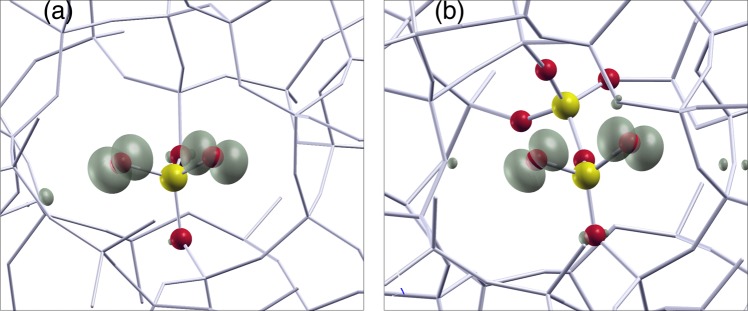
Spin-density (shadowed) calculated for the (**a**) model M2-I featuring an isolated [(O-)_2_P(=O)_2_]^0^ unit, and (**b**) two-corner sharing tetrahedra model M3-A of the r-POHC (*g*-tensor given in Table 3). Isolevel set at ~5% of grid maximum. For clarity only P and O atoms belonging to phosphate units are shown with yellow and red balls, while the surrounding silica network is shown with a stick model.

A calculation of the EPR parameters (Fermi contacts A_iso_(^31^P) and A_iso_(^17^O) and *g*-tensor principal values *g*_1_, *g*_2_, *g*_3_) has then been carried out for the positively charged and relaxed M2, M3, and M4 configurations. The spin-density of the configuration M3-A is shown in Fig. 5. The calculated values (4.5 to 4.8 mT) for the A_iso_(^31^P) of our r-POHC models are all in reasonable agreement with previous literature data, which reports a value of ~5 mT^29,35,59^. The two non-bridging oxygens show Fermi contacts of about 2.6 mT in our models. The calculated Fermi contacts are also in reasonable agreement with data available in the literature for POHC centers (1.7 to 4.9 mT)^35^. We note that a non negligible Fermi contact on P atoms can be found only for the P atom of the [(O-)_2_P(=O)_2_]^−^ unit. By contrast the P atom of the “companions” PO_4_ are EPR silent as their A_iso_(^31^P) are negligible (~0.1 mT). The bridging oxygen atoms are also silent (A_iso_ ~ 0.1 mT) thus making very difficult to directly infer the presence of a “companion” PO_4_ tetrahedron by means of EPR spectroscopy. The *g* values of both the M3 and M4 models are as good as than those calculated for the M2 model. As compared to the experimental *g*-values, the *g*_1_ of the M2 and M4 models is sligthly closer to the experiment than the *g*_1_ of the M3 models. *g*_2_ has the same value, in fair agreement with the experiments, for both models M2 and M3-A, and it is slightly smaller in M3-B and M4. A similar fair agreement with the experiments is also registered for the *g*_3_ which is slightly better in the M3-A than in the other models. However we note that the distribution of the *g*-values used to simulate the EPR spectra^29,35^, are quite large so that all models M2, M3, M4 can be regarded as good models of the r-POHC center as far as concerns the EPR parameters (Table 3). Moreover all of our models give *g*-values at least as good as those of the DFT-B3LYP calculations reported by Fanciulli *et al*.^35^

**Table 3:** *g*-tensor principal values and Fermi contacts A_iso_(P) and A_iso_(O)(mT) of the r-POHC: isolated (M2) vs corner-sharing PO_4_ tetrahedra models (M3, M4).

	*N*	*g*_1_	*g*_2_	*g*_3_	A_iso_(^31^P)	A_iso_(^17^O)
M2	0	2.0160	2.0105	2.0075	4.5	2.8
M3-A	1	2.0147	2.0105	2.0085	4.8	2.6
M3-B	1	2.0157	2.0097	2.0075	4.7	2.7
M4	2	2.0161	2.0093	2.0077	4.5	2.4
EXPT.^35^		2.0185	2.0115	2.0082	5.0	—
DFT^35^	0	2.0143	2.0097	2.0071	5.3	1.8

Expt. data and DFT (B3LYP) are taken from Fanciulli *et al*.^35^. *N* is the number of PO_4_ tetrahedra which are corner-sharing with the [(O-)_2_P(=O)_2_]^−^ unit.

## Discussion

The present work further confirms the assignment of the ~1145 cm^−1^ Raman band to symmetric stretching of P=O bonds in the [(O-)_2_P(=O)_2_]^−^ unit, as previously suggested by Fanciulli *et al*.^35^ the assignment of which, however, suffered from a ~50 cm^−1^ underestimation of the P=O symmetric stretching band. On the basis of our results, this underestimation should not be attributed to the size of the cluster model used by^35^. In fact, by using the same cluster model, we also computed a frequency (1117 cm^−1^) for the symmetric stretching of P=O bonds which again largely underestimates the position of the Raman band at ~1145 cm^−1^ ^38^. On the other hand, the Raman spectrum calculated for our glass model, containing an “ideal” isolated [(O-)_2_P(=O)_2_]^−^ unit (Fig. 3), supports in the glass a close frequency position (1112 cm^−1^) for the symmetric stretching of P=O as found in the cluster model. Taking into account the fact that our DFT models overestimate the mode frequencies around 1100 cm^−1^ in *v*-SiO_2_ by 20 cm^−1^, and even factoring in a potential underestimation of up to ~10 cm^−1^ (as for the P=O mode in M1), we estimate that in real P-doped silica glasses the symmetric stretching of P=O bonds in isolated [(O-)_2_P(=O)_2_]^−^ units should not occur above ~1120 cm^−1^. The discrepancy (~25 cm^−1^) with the position of the experimental Raman band at 1145 cm^−1^ is shown to be more easily explained by considering a [(O-)_2_P(=O)_2_]^−^ unit linked with at least one other phosphate tetrahedron (Models M3 and M4). The linking on one or on both sides with corner-sharing phosphate tetrahedra is sufficient to shift the P=O symmetric stretching (from ~20 to 40 cm^−1^) to higher frequencies, resulting in an improved agreement with the experimental data^35,38^. We also note that the ~40 cm^−1^ shift should be attributed to the shorter bond lengths^60^ (~0.01–0.02 Å) registered for the P=O bonds in M3 and M4 with respect to the ideal isolated [(O-)_2_P(=O)_2_]^−^ case as represented by the M2-I model.

Hence, the occurrence of the Raman band at ~1145 cm^−1^ may be considered as evidence in favor of the formation of “micro-clusters” or chains of few PO_4_ (at least two) connected tetrahedral units within the silica matrix. The size of these micro-clusters may be sensitive to the P-content, and so a small shift of the Raman band position could be expected for higher P doping, as the [(O-)_2_P(=O)_2_]^−^ unit is more likely to be connected with other phosphate tetrahedra of the P_2_O_5_ micro-cluster than with silica tetrahedra. Such a size-related sensitivity might explain the variation, in P-doped silica glasses, of the frequency position of the Raman band at ~1145 cm^−1^ which, in the literature, typically ranges between ~1140 cm^−1^ to 1160 cm^−1^ for P doping concentrations up to 30 mol%^17,35,36,38,39^. Furthermore the previous remarks are also consistent with assignment of the Raman band at about 1160 cm^−1^ in the Raman spectra of lithium and sodium ultraphosphate glasses to symmetric stretching of P=O bonds in the so-called Q^2^ phosphate tetrahedra with two bridging and two non-bridging oxygens^61,62^. However it should be noted that the large width ~100 cm^−1^ of the band at 1145 cm^−1^ is probably related to a quite large inhomogeneous broadening which could be explained by the occurrence of several kinds of configurations including all those here examinated (Figs 3 and 4). In particular in this work we have considered, among the possible precursors of the r-POHC, a P-doped configuration where an isolated [(O-)_2_P(=O)_2_]^−^ unit embedded silica relaxes by forming a penta-coordinated silicon, so that the P tetrahedron has only one non-bridging oxygen neighbor (model M2). On the basis of the calculated Raman spectrum, such a configuration would contribute to the Raman intensity around 1200 cm^−1^, and so it is likely to give only a minor contribution to the ~1150 cm^−1^ band. We verified that the network topology of the M2 final configuration does not depend on the choice of the energy functional adopted in the geometry optimization step. Hence we attribute the formation of the penta-coordinated silicon to the here adopted generation procedure (i.e. replacement of a twofold Si with a twofold P to which two extra non-bridging oxygen atoms are added by hand) which, depending on geometrical details e.g. the size of the void surrounding the twofold Si, might or might not allow for a proper first-principles relaxation of the isolated [(O-)_2_P(=O)_2_]^−^ unit (model M2-I). By contrast, we have shown that a [(O-)_2_P(=O)_2_]^−^ unit can be formed in P-doped silica, provided that a local seed of P_2_O_5_ composed by a few PO_4_ tetrahedra is present, as shown by the corner-sharing phosphate model discussed in the previous sections. Such a corner-sharing phosphate model allows for a successful interpretation of both the Raman band at ~1150 cm^−1^ and the occurrence of r-POHC centers in irradiated P-doped silica.

The precursor gas (POCl_3_) often used to produce P-doped silica via CVD techniques may react with oxygen leading to the formation of molecules of phosphorus oxide, namely P_4_O_10_ and P_2_O_7_. It is reasonable to assume that these molecules are often incorporated as block units^34,63^ and do not break apart when interacting with silica so that the usual way P is included in silica should not consist in one isolated PO_4_ tetrahedron, but rather in two (or more) corner-sharing PO_4_ tetrahedra included at a given point in the silica matrix. This should be the usual way to incorporate phosphorus atoms even for a P content lower than 5 mol%^23,64^. The formation of micro-clusters of P_2_O_5_ could be relevant also in the context of rare-earth doping since the rare-earth ions, e.g. Yb, are supposed to have a nearest-neighbor shell of phosphates and not be in contact directly with silica^20,21^. In fact, at the light of the present investigation, one may infer that P_2_O_5_ micro-clusters could easily and effectively surround the RE ion during the glass fabrication processes, even in case of low P doping content (~1 to 5 wt%). Yet, the formation of extended (mesoscopic or macroscopic) islands of P_2_O_5_ embedded in silica is ruled out by the fact e.g. that the stretching frequency of P=O bonds in P_2_O_5_ occurs at higher frequencies^56^ i.e. 1390 cm^−1^ with respect to P-doped silica (~1320–1330 cm^−1^), and for P content up to 30 mol% only the latter has been observed^35,38,58^.

Previous experimental Raman investigations^34,36,39,58^ have found Raman bands near ~1330, ~1150, ~1025, ~715, ~620 and ~530 cm^−1^. A detailed investigation of the origin of Raman bands in P-doped silica is out of scope of the present paper. However we note that the Raman spectra of all the models here investigated show an increased intensity at about 500 cm^−1^ and in the range ~650–800 cm^−1^, which are the typical frequencies of the bending modes of phosphate tetrahedra.

In conclusion, in this work we have discussed two alternative models of the r-POHC. The first one, based on the occurrence of an isolated [(O-)_2_P(=O)_2_]^−^ unit embodied in the silica glass matrix, is shown to be consistent with EPR data, but as far as concerns Raman data, the symmetric stretching of P=O bonds is shown to be compatible with a band located not above ~1120 cm^−1^, and so does not clearly explain the experimental feature observed at ~1145 cm^−1^, The second model considered here consists in a [(O-)_2_P(=O)_2_]^−^ unit, corner-sharing with at least one phosphate tetrahedron, incorporated in pure silica. EPR and Raman calculations based on such a model agree fairly well the experimental results. Hence the present work strongly suggests that the precursor site of a r-POHC is likely to be constituted by a micro-cluster of a few (at least two) neighboring phosphate tetrahedra, and also suggests that occurrence of isolated [(O-)_2_P(=O)_2_]^−^ units is unlikely even at low P-doping concentrations. In particular, at variance with the isolated model of the [(O-)_2_P(=O)_2_]^−^, only the corner-sharing model allows to calculate a frequency for the symmetric stretching of P=O bonds that is in good agreement with the location of the experimental Raman band at ~1145 cm^−1^. Hence the latter Raman band has to be regarded as a fingerprint of the presence of corner-sharing phosphate tetrahedra (P_2_${{\rm{O}}}_{7}^{-}$) even at low P content. This fact might be relevant also in explaining conversion mechanisms as well as luminescence properties of point defects in P-doped glasses^25,26,63^. Furthermore, the present results could pave the way for the development of new codoping strategies aiming at separating phosphate tetrahedra so to reduce the number of generation and conversion processes leading to the formation of P_1_ centers which are the main responsible for the attenuation of the signal at wavelength of 1550 nm in high power optical fiber amplifiers^27^.

## Methods

The calculations carried out in this work are based on density functional theory (DFT). In particular, the local density approximation (LDA) exchange-correlation functional has been adopted for the DFT calculations included in this work^65^. LDA calculations are perfectly suitable to study vibrational properties of silica based materials^42^, and notably as far as concerns the frequencies related to Si-O stretching (~1000–1200 cm^−1^). We note that the use of more expensive hybrid functionals does not guarantee an improved result with respect to other less expensive DFT approaches. For instance, a vibrational density of states calculated using the B3LYP functional underestimated by 20–30 cm^−1^ the position of the main peaks in the Si-O stretching region^44^. Norm-conserving Trouiller-Martins and Bachelet-Hamann-Schlüter pseudopotentials are used for O and for P and Si atoms respectively^66,67^. Kohn-Sham wavefunctions are expanded in a basis of plane waves up to a kinetic cutoff of 70 Ry. The wavefunctions were expanded at the sole Γ point of the Brillouin zone, as justified by the large size and the large band gap of our system. Geometry optimizations have been obtained by means of non-spin-polarized calculations, though spin-polarized calculations (occupations of states are fixed to be either 1 or 0) were performed to test a few hypothesis as reported in the P-doped models section by using a generalised gradient approximation (GGA) functional [i.e. the Perdew-Burke-Ernzerhof (PBE) functional]^41^. PBE0 hybrid functional^68^ was also used to carry out dedicated structural relaxations. The adopted exx fraction is 32.5% which provides for silica models a major correction (~4 eV) of the band gap with respect to standard DFT functionals.

Calculation of the vibrational modes have been performed by using a linear response approach^69,70^. In particular we have taken care that no negative vibrational frequencies are obtained i.e. parameters used in the structural optimization (force threshold 0.0025 eV/Å) allow for a proper harmonic treatment. The Raman cross section has been calculated assuming non-resonant conditions in the Placzek approximation as described in a previous work^42^. The codes used for the present calculations of structural and vibrational properties are freely available with the Quantum-Espresso (QE) package v6.0^71^. The EPR parameters are calculated at the GGA level^41^ by exploiting the gauge including projector augmented wave (GIPAW) method as available in the QE package^72^.

## Supplementary information

Supplementary information file

## Data Availability

The datasets generated during and/or analysed during the present study are available from the corresponding author on reasonable request.
