# Supplementary material for: v-P2O5 micro-clustering in P-doped silica studied by a first-principles Raman investigation
Source: Sci Rep. 2019 May 9;9:7126. doi: 10.1038/s41598-019-42887-3 (PMC6509213; doi:10.1038/s41598-019-42887-3)
Supplement: Supplementary file 1 — Supplementary information file [file 41598_2019_42887_MOESM1_ESM.pdf]

v-P_2O_5 micro-clustering in P-doped silica studied by a first-principles Raman investigation

Luigi Giacomazzi[1,2,*], L. Martin-Samos[2], A. Alessi[3], N. Richard[4], A. Boukenter[3], Y. Ouerdane[3], S. Girard[3], M. Valant[1], and S. De Gironcoli[5]

[1]MaterialsResearch Laboratory, University of Nova Gorica, Vipavska 11c 5270-Ajdovščina, Slovenia.

[2]CNR-IOM/Democritos National Simulation Center, Istituto Officina dei Materiali, c/o SISSA, via Bonomea 265, IT-34136 Trieste, Italy.

[3]Univ Lyon, UJM-Saint-Etienne, CNRS, IOGS, Laboratoire Hubert Curien UMR 5516, F-42023 St-Etienne, France.

[4]CEA, DAM, DIF, F-91297 Arpajon, France.

[5]SISSA via Bonomea 265, IT-34136 Trieste, Italy.

*corresponding author, E-mail: lgiacomazzi@ung.si

Supplementary Information:

Supplementary Figures:

Supplementary Figure S1: Participation ratio of vibrational modes as calculated for the original pure silica model (circles) and as calculated for the P-doped models M1 (squares) and M2-I (discs).

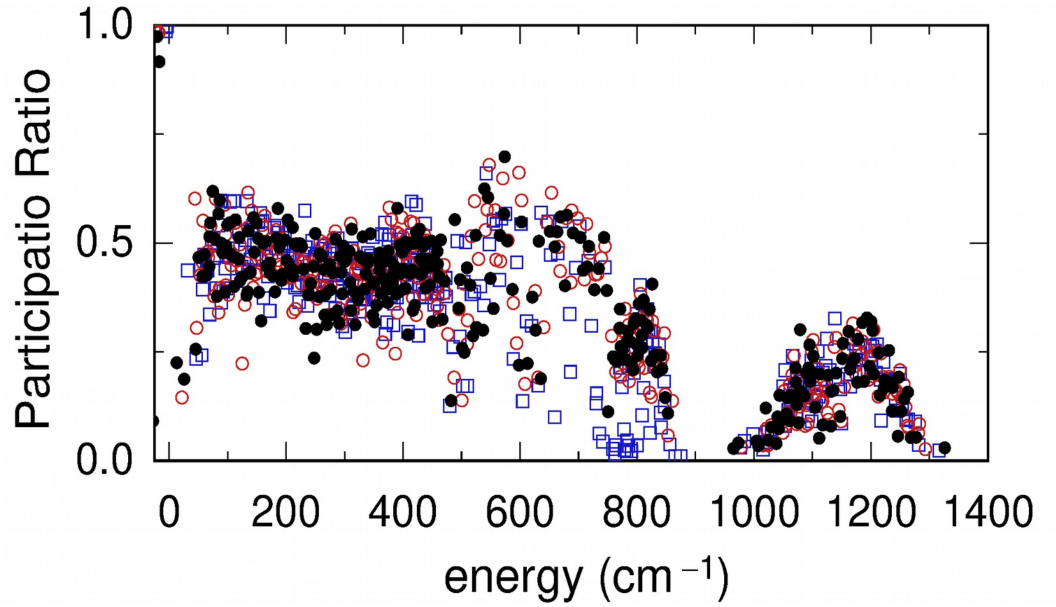

The participation ratio [R.J. Bell *et al*, J. Phys. C: Solid St. Phys. **3**, 2111 (1970)] of modes in the region 1000 to 1300 cm^{-1} (~0.2) indicates a rather high degree of localization (a fully delocalized mode has a participation ratio of ~1, while a participation ratio less than ~0.1 indicates localization on a few atoms.

Supplementary Figure S2: Raman spectrum of the silica model including a substitutional P atom (belonging to a 3-fold ring and to a 4-fold ring) compared to the original pure silica model.

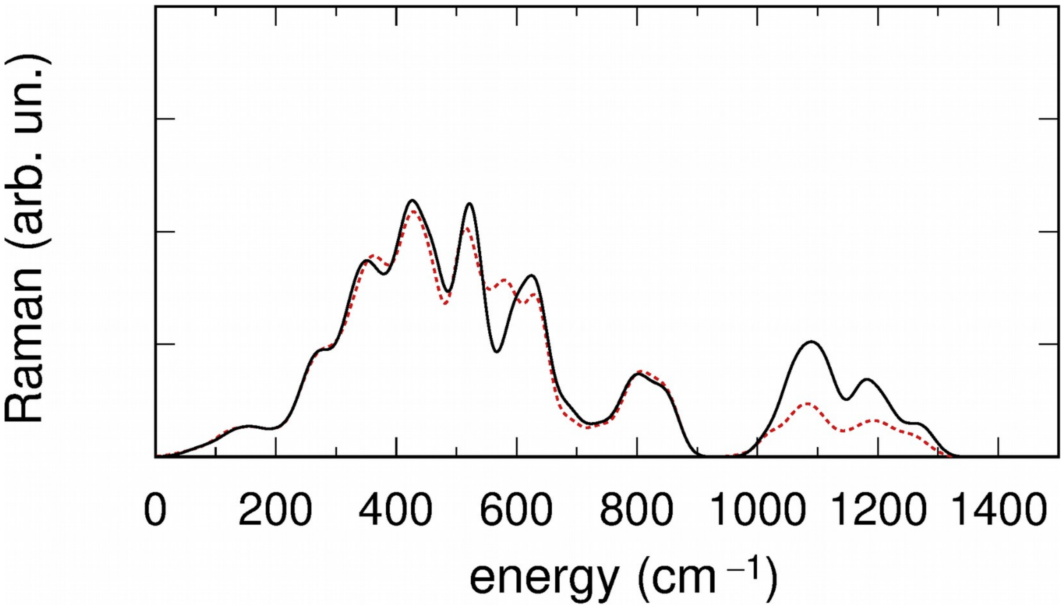

The inclusion of a substitutional P_{Si} (i.e. a PO_4^+) mainly affects the Raman intensity of the stretching region (900-1300 cm^{-1}) with an increase of a factor about 2, but with no feature visible at ~1150 cm^{-1} . The increase of intensity in the stretching region is a consequence of the fact that by including substitutional P atoms, highly Si-O stretched bonds (1.68 Å) are formed for the O belonging to the P-O-Si bridge, which do show a considerable larger Raman polarizability (in the stretching direction) with respect to standard bridging oxygen atoms in Si-O-Si bridges.

Supplementary Figure S3: Vibrational mode at 650 cm^{-1} in the M1 model, i.e. of a [(O-)$_3$P=O] unit in silica showing the antisymmetric bending vibrational mode of the phosphate tetrahedron (P, Si, O atoms are shown with purple, yellow, and red balls).

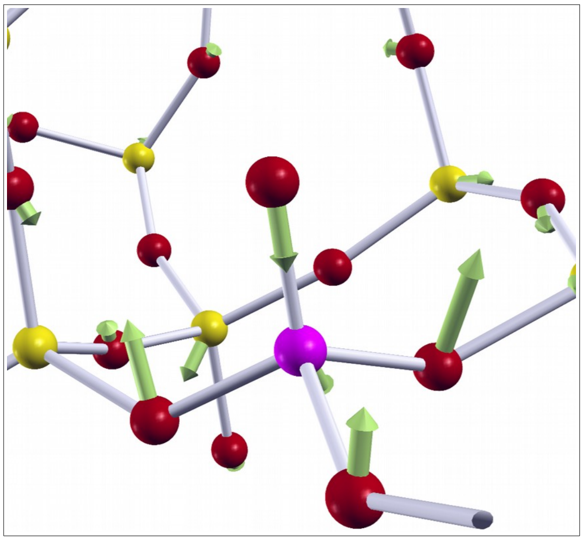

Supplementary Figure S4: Vibrational mode at about 1220 cm^{-1} in the model M2, showing displacements of oxygen atoms belonging to the pentacoordinated silicon connected to the phosphate unit.

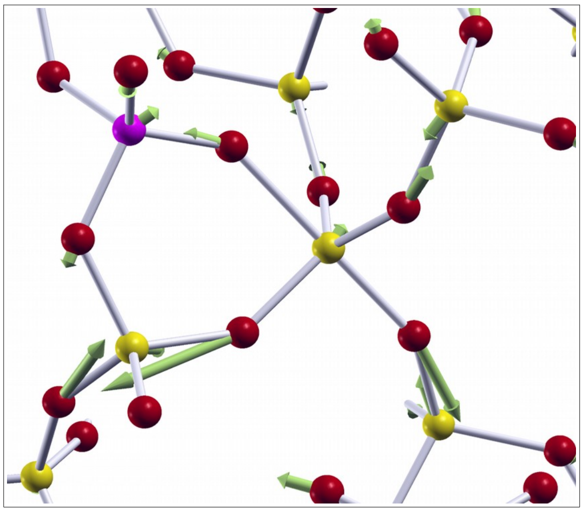

Supplementary Figure S5: Raman intensities calculated for the vibrational modes of the cluster $O_2P(OSiO_3H_3)_2$

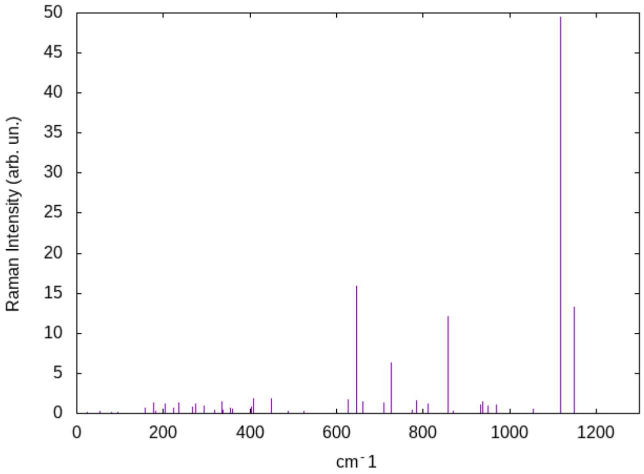

The symmetric stretching of P=O bonds occur at 1117 cm^{-1} and 1150 cm^{-1}, with the largest intensity occurring at 1117 cm^{-1} (about four times more than at 1150 cm^{-1})
